# Web-Based Self-Assessment Health Tools: Who Are the Users and What Is the Impact of Missing Input Information?

**DOI:** 10.2196/jmir.3146

**Published:** 2014-09-26

**Authors:** Nicole Neufingerl, Mark R Cobain, Rachel S Newson

**Affiliations:** ^1^Nutrition & Health DepartmentUnilever Research & DevelopmentVlaardingenNetherlands; ^2^New Business UnitUnilever Research & DevelopmentLondonUnited Kingdom

**Keywords:** cardiovascular disease, risk assessment, Web applications, consumer health information, preventive health services, cholesterol, blood pressure

## Abstract

**Background:**

Web-based health applications, such as self-assessment tools, can aid in the early detection and prevention of diseases. However, there are concerns as to whether such tools actually reach users with elevated disease risk (where prevention efforts are still viable), and whether inaccurate or missing information on risk factors may lead to incorrect evaluations.

**Objective:**

This study aimed to evaluate (1) evaluate whether a Web-based cardiovascular disease (CVD) risk communication tool (Heart Age tool) was reaching users at risk of developing CVD, (2) the impact of awareness of total cholesterol (TC), HDL-cholesterol (HDL-C), and systolic blood pressure (SBP) values on the risk estimates, and (3) the key predictors of awareness and reporting of physiological risk factors.

**Methods:**

Heart Age is a tool available via a free open access website. Data from 2,744,091 first-time users aged 21-80 years with no prior heart disease were collected from 13 countries in 2009-2011. Users self-reported demographic and CVD risk factor information. Based on these data, an individual’s 10-year CVD risk was calculated according to Framingham CVD risk models and translated into a Heart Age. This is the age for which the individual’s reported CVD risk would be considered “normal”. Depending on the availability of known TC, HDL-C, and SBP values, different algorithms were applied. The impact of awareness of TC, HDL-C, and SBP values on Heart Age was determined using a subsample that had complete risk factor information.

**Results:**

Heart Age users (N=2,744,091) were mostly in their 20s (22.76%) and 40s (23.99%), female (56.03%), had multiple (mean 2.9, SD 1.4) risk factors, and a Heart Age exceeding their chronological age (mean 4.00, SD 6.43 years). The proportion of users unaware of their TC, HDL-C, or SBP values was high (77.47%, 93.03%, and 46.55% respectively). Lacking awareness of physiological risk factor values led to overestimation of Heart Age by an average 2.1-4.5 years depending on the (combination of) unknown risk factors (*P*<.001). Overestimation was greater in women than in men, increased with age, and decreased with increasing CVD risk. Awareness of physiological risk factor values was higher among diabetics (OR 1.47, 95% CI 1.46-1.50 and OR 1.74, 95% CI 1.71-1.77), those with family history of CVD (OR 1.22, 95% CI 1.22-1.23 and OR 1.43, 95% CI 1.42-1.44), and increased with age (OR 1.05, 95% CI 1.05-1.05 and OR 1.07, 95% CI 1.07-1.07). It was lower in smokers (OR 0.52, 95% CI 0.52-0.53 and OR 0.71, 95% CI 0.71-0.72) and decreased with increasing Heart Age (OR 0.92, 95% CI 0.92-0.92 and OR 0.97, 95% CI 0.96-0.97) (all *P*<.001).

**Conclusions:**

The Heart Age tool reached users with low-moderate CVD risk, but with multiple elevated CVD risk factors, and a heart age higher than their real age. This highlights that Web-based self-assessment health tools can be a useful means to interact with people who are at risk of developing disease, but where interventions are still viable. Missing information in the self-assessment health tools was shown to result in inaccurate self-health assessments. Subgroups at risk of not knowing their risk factors are identifiable and should be specifically targeted in health awareness programs.

## Introduction

 During the last decade, access to the Internet and the proportion of the population using it to seek health information have markedly increased [1,2]. Online health information seekers are typically searching for information regarding a specific disease or medical problem, including the potential to diagnose their own health status [3,4]. Health professionals, public health and governmental organizations, and private health providers are therefore using the Internet as a medium to disseminate health information and preventative educational programs. This makes the Internet a valuable instrument for increasing consumer awareness, promotion of healthy behaviors, and disease prevention [5,6]. Web-based health applications, such as self-assessment health tests or behavior change programs, that combine high-quality health information with interactive components can therefore play a role to benefit prevention, early detection, or treatment of non-communicable diseases [7-9].

The impact that Web-based health assessment tools can have on public health depends on the audience they reach, as well as on the quality and reliability of the information they provide. Criticism of Web-based health applications has been raised suggesting that they may not be reaching the people who need these tools the most: those at risk of developing disease, and where prevention efforts are still viable [10,11]. Furthermore, there is concern about the potential harm that can be caused by inaccurate health assessments provided on the Internet that can deliver incorrect diagnoses and/or cause delays in seeking appropriate medical care [12,13]. This can arise due to poorly designed Web applications that are not based on scientific evidence. Alternatively, inaccurate health assessment results may arise from users providing inaccurate information due to inputting the information incorrectly, not understanding questions, or they may not know the answer to the questions [14]. This may be particularly pertinent to tools that require information on physiological risk factors, which are unknown to a large part of the population [5,15].

The potential impact of Web-based health assessment tools on disease prevention is large, but they need to reach users with an elevated disease risk and provide accurate health assessments. Therefore, the aim of the current study was to (1) evaluate whether a Web-based health assessment tool communicating cardiovascular disease (CVD) risk was reaching users at risk of developing CVD, (2) evaluate the impact of awareness of physiological risk factors on the health assessment provided by the CVD risk communication tool, in particular total cholesterol (TC), HDL-cholesterol (HDL-C), and systolic blood pressure (SBP), and (3) evaluate the key predictors of awareness and reporting of physiological risk factors in order to understand who in the general population is more likely to provide physiological measures to Web-based tools.

## Methods

### Study Design

The current study was based on a global database from users of the Heart Age tool. Heart Age was developed to help users better understand their risk of CVD, which when given as a traditional percentage can be a difficult concept to understand (eg, “your risk of a CVD event in the next 10 years is 12%”) [16,17]. Heart Age is a Web-based tool that obtains an individual’s CVD risk factor information through a series of questions, calculates percentage risk of developing CVD within the next 10 years according to the Framingham risk score, and then translates this risk into a “Heart Age” [18]. An individual’s Heart Age corresponds to the age of a person with the same predicted CVD risk but with all other risk factors considered as normal. This means that depending on their CVD risk, a person’s Heart Age could be younger or older than their chronological age. For example, a 55-year-old man with normal risk factors would have a 10% risk of CVD; accordingly, a 40-year-old man with a 10% risk of CVD (due to unhealthy risk factors) would have a Heart Age of 55 years. The Heart Age tool aims to make users aware of their risk of developing a CVD event and which risk factors are contributing to this risk, in order to motivate them to make lifestyle changes. Therefore the target audience is anyone at risk of developing a CVD event, but where prevention efforts are still viable, as these users can benefit the most from awareness of their current status and potential interventions. After completion of the Heart Age test, users can sign up for a free Web-based health program providing personalized diet and lifestyle advice to lower one’s Heart Age. The Heart Age tool is directed at the general population, but in particular it intends to reach users with unhealthy risk factors. Heart Age was launched globally in 2009 as part of a brand marketing campaign by Flora/Becel, a margarine brand, via on-pack messages, advertisements on television, newspapers, in store applications, etc. The campaign focused on raising awareness of heart health; it was not targeted at specific at risk groups. The tool is available via free open access websites available in different languages [19].

### Users

Data from 3,374,769 users of the Heart Age tool was collected between July 2009 and December 2011. The tool was launched in 14 countries, including United Kingdom, Germany, the Netherlands, Belgium, Finland, Austria, Poland, Turkey, Ireland, Portugal, Slovenia, Greece, Australia, and Brazil. By using the tool, users consented to the privacy policy, which could be accessed via a link on the website, and which stated that personal information provided could be collected and used in an aggregated way to evaluate the use of the site and services provided. Visitors to the website did not receive any incentives for using the tool. All of the information users entered was stored in a format where individuals could not be identified. The use of Heart Age was restricted to users aged between 20 and 80 years old with no history of heart disease. This group was selected because this was the population on which the original CVD algorithms were validated, and people with existing heart disease have different levels of risk of a future CVD event. A disproportionate number of users of the Heart Age tool reported to be 20 years old (9.59%, 323,547/3,374,769), possibly because this was the default setting. Therefore, to prevent artificial overrepresentation of 20 year olds, analysis was restricted to users aged 21-80 years.

To identify repeat users, prior to starting the self-assessment users were asked to indicate whether they had used the tool before. All return users were excluded from analysis to prevent duplication (n=143,682). Another 32,700 users whose TC, HDL-C, SBP, and body mass index (BMI) values were all missing or beyond defined valid ranges based on clinical judgment (ie, 80 mmHg≤ SBP ≤220 mmHg; 77 mg/dl≤ TC ≤423 mg/dl; 25 mg/dl≤ HDL ≤90 mg/dl; 15≤ BMI ≤45) were excluded from analysis as it was not possible to calculate a Heart Age for these users. As the tool was never promoted in Brazil and the number of users from Brazil was very low (n=102), it is likely that most entries were from a select group of internal users; therefore, all Brazilian users were excluded from the analysis. Finally, 130,647 users were excluded based on identical data of date of using the tool, BMI, TC, HDL, SPB, Heart Age, and 10-year CVD risk, which was mainly due to testing the functionality of the tool during the developmental phase. This resulted in a final dataset of 2,744,091 users. Among those users who were excluded due to reasons other than being 20 years old (n=307,137), the mean age was 40.98 (SD 14.29) years and 50.78% (155,969/307,137) were women.

### Materials

The Heart Age tool assessed CVD risk factors by self-report. Users completed a Web-based questionnaire that asked for information on demographic data (ie, age, gender, height, and weight; family history of CVD), physiological measures (ie, TC, HDL-C, and SBP values), other CVD risk factors (ie, smoking status and diabetes prevalence), and use of antihypertensive or cholesterol lowering medication. Further information was also obtained relating to CVD disease but was not relevant to the aims of the current study.

**Figure 1:**
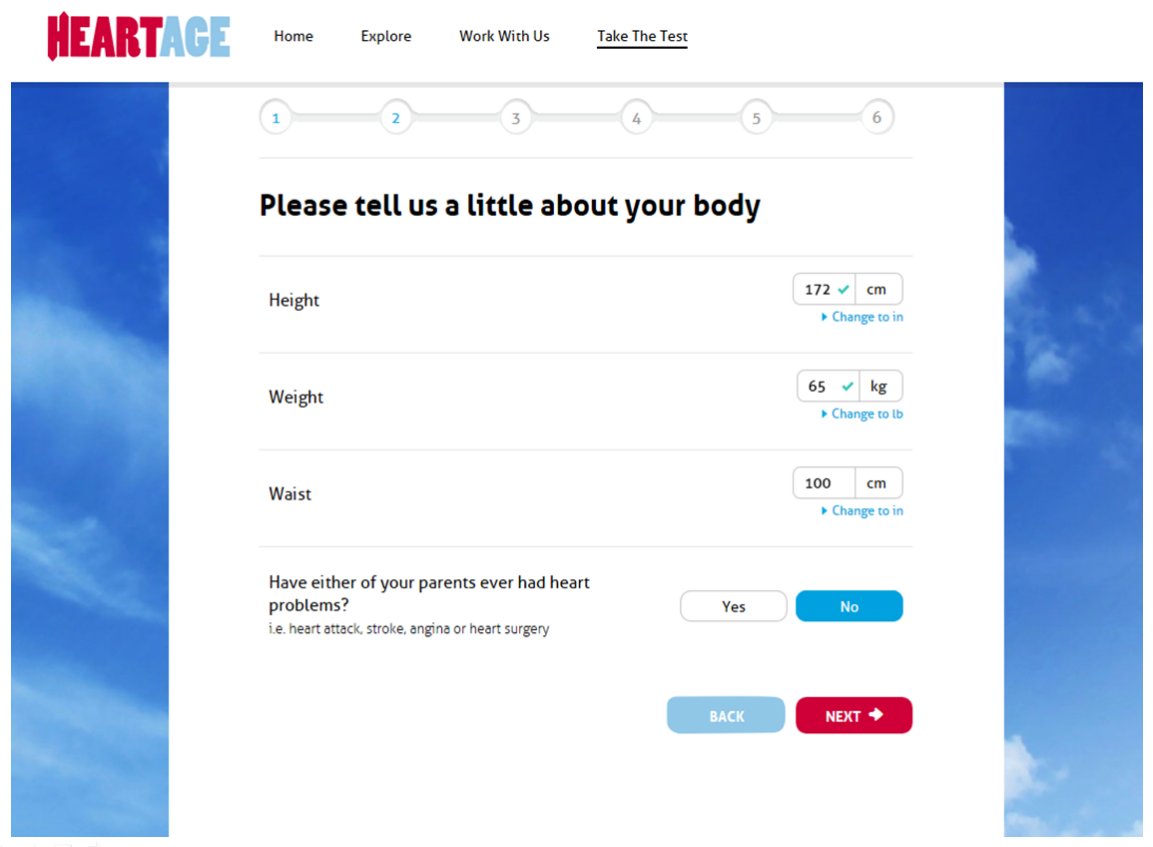
Screenshot of the Heart Age tool.

Questions were mostly presented as closed questions with locked answer options (eg, “yes/no” or “male/female”). Family history of CVD was assessed by asking “Have either of your parents ever had heart problems (ie, heart attack, stroke, angina, or heart surgery?)”. Smoking status and diabetes prevalence were determined by asking “Do you smoke?” and “Do you have diabetes?” Use of antihypertensive and blood cholesterol lowering medication was assessed with the questions “Are you taking or have you ever taken medication to lower your blood pressure?” and “Are you taking or have you ever taken medication to lower your cholesterol?” Questions on age, height, weight, and physiological measures were asked using an open answer format. Users could choose in which unit they wanted to enter their values for height (cm or inch) and weight (kg or lb); units for cholesterol were pre-determined and differed per country (mmol/l or mg/dl). Physiological measures were assessed by asking “Do you know your cholesterol level?” and “Do you know your blood pressure?” If users indicated “yes”, a sub-dialogue box opened and they could enter a value for TC and HDL-C or for SBP, respectively. The sub-dialogue boxes also contained explanations stating that “HDL cholesterol is sometimes known as ‘good’ cholesterol” and that “Systolic blood pressure is the higher number (eg, where blood pressure is 120/80, 120 is the systolic reading)”. All subject data were automatically captured in a database. See Figure 1 for a screenshot of the Heart Age tool. Screenshots of the complete user journey through the Heart Age tool are provided in Multimedia Appendix 1.

Depending on the availability of the physiological measures per individual, 10-year CVD risk and Heart Age could be calculated using different algorithms. There was one full algorithm when all physiological measures were known, and 5 alternatives for when one or more of the physiological measures were missing. The following alternative algorithms were available: (1) TC & HDL, (2) TC, SBP, BMI, (3) TC, BMI, (4) SBP, BMI, and (5) BMI. The CVD algorithms were developed using Framingham cohort data as sex-specific multivariable risk functions based on Cox proportional-hazards regression. The full CVD risk algorithm included data on age, gender, TC, HDL-C, SBP, antihypertensive medication use, smoking status, and diabetes [18]. Detailed information about the development and validation of the algorithms to calculate 10-year CVD risk can be found elsewhere [20,21]. Once calculated, CVD risk was translated into a Heart Age [20]. An individual’s Heart Age corresponds to the age of a person with the same predicted CVD risk but with all other risk factors considered as “normal”. The reference values for “normal” risk factors in the full Heart Age algorithm, were defined as not smoking, not diabetic, SBP=125 mmHg (130 mmHg if 60 or above), TC=180 mg/dl, and HDL-C=45 mg/dl [18]. For the alternative models where total cholesterol was present without HDL-C, the reference value for a person with “normal cholesterol” was increased to 200 mg/dl to increase the sensitivity of these models. To keep Heart Ages within a reasonable range, it was decided that Heart Age should be capped if it was 15 years lower or higher than chronological age, or if it fell below 18 or exceeded 80 years of age, in order to alert people of the need for change and medical advice without creating alarm. An overview of all CVD and Heart Age algorithms is provided Multimedia Appendix 2.

### Data Analysis

Physiologic measures were analyzed both as continuous variables and dichotomous variables (normal vs high or normal vs low). Users were classified as having high TC if TC≥240 mg/dl, low HDL-C if HDL-C≤40 mg/dl [22], and high SBP was defined as SBP≥140 mmHg [23]. BMI was calculated from self-reported data on height and weight, and overweight and obesity were defined as BMI≥25 kg/m^2^ and BMI≥30 kg/m^2^ respectively [24]. All values beyond valid ranges were considered missing and treated as case-wise missing in analyses. To evaluate Heart Age data independent of chronological age, the difference between Heart Age and chronological age was calculated as “relative Heart Age”.

To evaluate the effect of awareness of TC, HDL-C, and/or SBP values on Heart Age, a subsample was used consisting of users with valid values available on all physiological measures (173,397/2,744,091; 6.32% of the total population). For the users in this subsample, we calculated Heart Age based on the full algorithm and each of the five alternative algorithms. Repeated measures analysis of covariance (ANCOVA) were then conducted to test for significant differences in relative Heart Age between the full and alternative algorithms, adjusting for the effects of age, gender, and country. To evaluate if there were differential associations based on CVD risk category, age, and gender (ie, factors strongly associated with CVD risk), univariate ANCOVAs were conducted stratifying for CVD risk category, age, and gender. To account for multiple comparisons, *P* values in post-hoc tests were adjusted using a Bonferroni correction. Results are presented per gender, 10-year age categories, and 10-year CVD risk categories based on the Framingham risk score [18].

To evaluate the associations between subject characteristics and awareness of the three physiological cardiovascular risk factor values, logistic regression analyses were conducted based on the total sample. A separate regression model for each characteristic was conducted (ie, demographics, risk factors, CVD risk estimates, physiological measures (continuous and categorical, and awareness thereof), controlling for the effect of age, gender, and country.

##  Results

### Characteristics of Users of the Web-Based Tool

Of the 2,744,091 Heart Age users included in the data analysis, the majority were from United Kingdom (31.19%, n=855,822), Germany (18.35%, n=503,502), the Netherlands (18.33%, n=503,063) Belgium (15.01%, n=411,948), and Finland (9.18%, n=251,952). The other countries contributed with 0.04% (n=995) to 2.33% (n=64,050) to the studied sample. The age distribution of Heart Age users covered the complete spectrum from 21-80 years of age. The number of users in their early 20s was high and peaked again between 40 and 50 years of age. Of the total sample of 2,744,091 users, 22.76% (n=624,639) were 20-29 years old, and another 23.99% (n=658,357) were 40-49 years old. After age 60, the number of Heart Age users decreased sharply, with 10.92% of users (n=299,665) aged 60-69 years and only 2.94% of users (n=80,753) aged 70-80 years.

The demographic characteristics and risk factor profile of the sample are presented in Table 1. On average, users had a Heart Age that exceeded their chronological age and reported multiple unhealthy CVD risk factors. The physiologic measures were normally distributed across the sample with mean values for TC and HDL in the normal range and for SBP and BMI above the normal range. Almost one third of the 2,744,091 users reported having a family history of CVD (32.84%, n=901,134) and prevalence of diabetes was low (3.39%, n=93,020). Heart Age was used more often by women (56.03%, n=1,537,490) than by men. Female users of the tool had on average a lower CVD risk and a lower relative Heart Age than men, a lower prevalence of modifiable risk factors, and more favorable physiological risk factor values, with the exception of TC. Interestingly, family history of CVD was more frequent among female users (35.70%, n=548,949) than male users (29.19%, n=352,185). Compared to the total sample, users with valid values for all physiologic measures and BMI were older. There were also more diabetic people, people with a family history of CVD, and fewer smokers in this subsample of users. Mean TC, HDL-C, and SBP values were similar between the total and subsample. Users in the subsample had fewer unhealthy CVD risk factors than the total sample. While the 10-year CVD risk of users with valid values for all physiologic measures and BMI was higher compared to that of the average user (due to older chronological age), the average user had a higher relative Heart Age than the users in the subsample.

**Table 1:** Characteristics of users of the Heart Age tool, per gender and for a subsample of users with complete valid values for TC, HDL-C, and SBP.

Characteristics		Total sample (N=2,744,091)	Men (n=1,206,601)	Women (n=1,537,490)	Subsample (n=173,397)
**Demographics**					
	Age in yrs, mean (SD)	42.9 (14.0)	42.9 (14.3)	42.9 (13.8)	52.5 (12.0)
	Male gender, n (%)	1,206,601 (43.97)	1,206,601 (100.00)	0 (0.00)	85,107 (49.08)
	BMI (m/kg^2^), mean (SD)^a^	25.9 (4.8)	26.3 (4.3)	25.6 (5.2)	26.0 (4.3)
**CVD risk estimate, mean (SD)**					
	Heart Age, yrs^b^	46.9 (16.3)	47.8 (16.2)	46.2 (16.4)	53.6 (15.3)
	Heart Age minus age, yrs^b^	4.0 (6.4)	4.9 (5.9)	3.3 (6.7)	1.2 (8.5)
	10-yr CVD risk, %	7.15 (9.03)	10.30 (11.26)	4.69 (5.66)	9.90 (9.59)
	Total unhealthy CVD risk factors, n	2.9 (1.4)	3.1 (1.4)	2.8 (1.4)	1.9 (1.4)
**Risk factor prevalence, n (%)**					
	Current smoker	641,338 (23.37)	314,452 (26.06)	326,886 (21.26)	20,253 (11.68)
	Overweight, BMI≥25^a^	1,391,691 (50.74)	693,071 (57.46)	698,620 (45.46)	91,721 (52.90)
	Obese, BMI≥30^a^	475,715 (17.34)	201,322 (16.69)	274,393 (17.86)	26,525 (15.29)
	Diabetic	93,020 (3.39)	49,577 (4.11)	43,443 (2.83)	12,448 (7.18)
	High TC (≥240 mg/dL)^c^	87,257 (14.11)	35,624 (13.00)	51,633 (15.00)	23,432 (13.51)
	Low HDL-C (≤40 mg/dL)^d^	31,067 (16.25)	20,616 (22.07)	10,451 (10.68)	27,881 (16.08)
	High SBP (≥140 mmHg)^e^	116,836 (7.97)	60,506 (9.75)	56,330 (6.66)	13,716 (7.91)
	Family history of CVD	901,134 (32.84)	352,185 (29.19)	548,949 (35.70)	76,919 (44.36)
**Physiologic measures, mean (SD)**					
	TC (mg/dL)^c^	195.9 (47.0)	193.5 (44.0)	197.7 (46.6)	195.5 (44.6)
	HDL-C (mg/dL)^d^	58.5 (15.6)	61.6 (16.4)	62.5 (15.1)	58.6 (15.6)
	SBP (mmHg)^e^	124.6 (14.4)	127.1 (13.3)	122.4 (14.6)	126.1 (13.0)

^a^Users with valid BMI values (n=2,742,818 in total sample; 1,206,092 men and 1,536,726 women).

^b^Heart Age was calculated from different algorithms depending on available valid values of TC, HDL-C, and SBP.

^c^Users with valid TC values (n=618,210 in total sample; 274,102 men and 344,108 women).

^d^Users with valid HDL-C values (n=191,219 in total sample; 93,380 men and 97,839 women).

^e^Users with valid SBP values (n=1,466,836 in total sample; 620,684 men and 846,152 women).

### Awareness of Physiological Risk Factors

The majority of Heart Age users were not aware of their TC values (77.47%; n=2,125,881) or HDL-C values (93.03%; n=2,552,872). Awareness of SBP values was relatively the highest, with 53.45% (n=1,466,836) of the users reporting valid values. Of all users, 6.37% (n=174,670) reported valid values for all three physiological risk factors.

### Estimated Impact of Awareness of Physiological Risk Factors on Heart Age (in Subsample With Complete CVD Risk Factor Values)

Relative Heart Age, as calculated according to the five alternative algorithms, was compared to the relative Heart Age calculated with the full algorithm. This was done among the subsample of users with complete valid values for all physiologic measures and BMI to enhance comparability across algorithms. Alternative algorithms generally overestimated Heart Age, resulting in a significantly higher relative Heart Age than the full algorithm (*P*<.001). Alternative algorithms that were based on two physiological measures were closer to the full algorithm than alternative algorithms with only one, or no, physiological measure. The mean differences in relative Heart Age between the alternative and full models, adjusted for age, gender, and country, was 2.1 years (SD 0.1) for the alternative algorithm #1 (TC & HDL-C), 2.2 years (SD 0.1) for alternative algorithm #2 (TC, SBP, & BMI), 3.0 years (SD 0.1) for alternative algorithm #4 (SBP & BMI), and 3.9 years (SD 0.1) for alternative algorithm #3 (TC & BMI). The alternative algorithm #5, which included BMI as the only physiological measure, deviated most from the full algorithm, with on average 4.5 (SD 0.1) years higher than the full algorithm.

**Figure 2:**
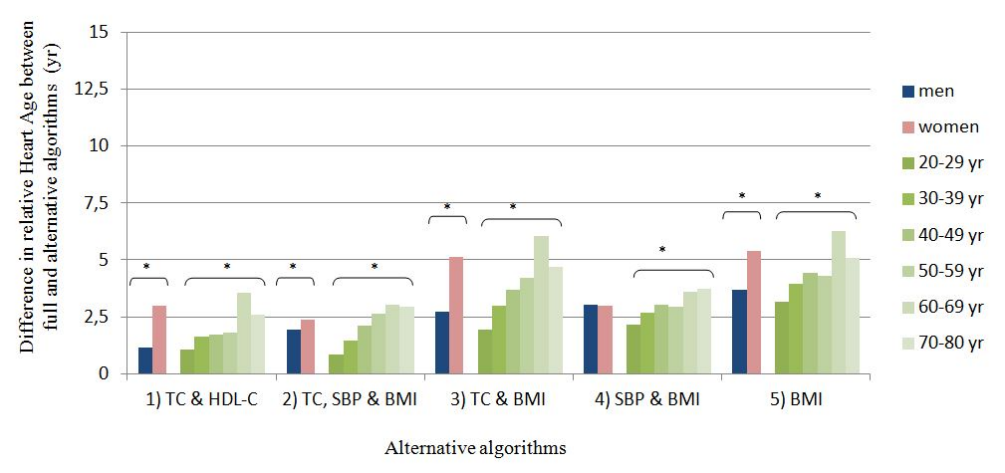
Mean difference in relative Heart Age (i.e. the difference between users’ Heart Age and chronological age) between the full and alternative algorithms per gender and age category. The analysis was restricted to a sub-sample of users with complete valid values for total cholesterol (TC), HDL-cholesterol (HDL-C), systolic blood pressure (SBP) and BMI (n=173,397), and adjusted for age, gender and country where appropriate. Means are estimated marginal means. * significantly different, p < 0.001.

**Figure 3:**
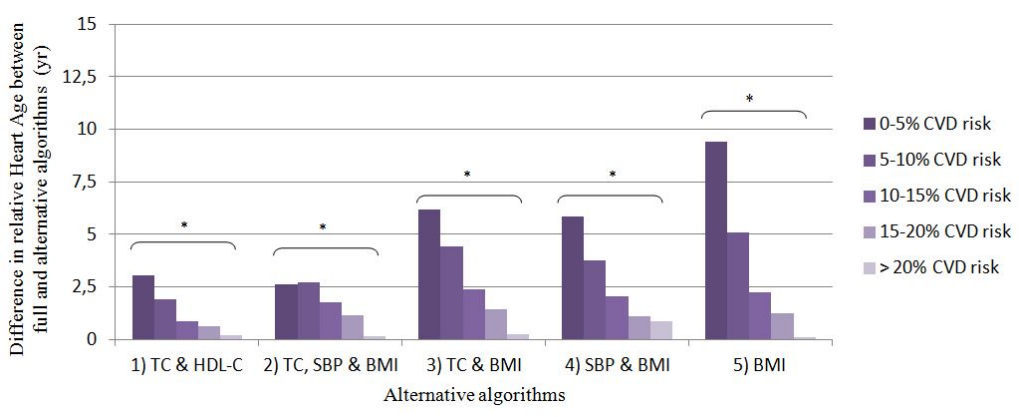
Mean difference in relative Heart Age (i.e. the difference between users’ Heart Age and chronological age) between the full and alternative algorithms per category of absolute 10 year CVD risk based on Framingham risk score [18]. The analysis was restricted to a sub-sample of users with complete valid values for total cholesterol (TC), HDL-cholesterol (HDL-C), systolic blood pressure (SBP) and BMI (n=173,397), and adjusted for age, gender and country where appropriate. Means are estimated marginal means. * significantly different, p < 0.001.

ANCOVAs comparing the difference in relative Heart Age between the full and all alternative algorithms revealed significant effects of age, gender, and CVD risk category (*P*<.001). These analyses were also restricted to the subsample of users with complete valid values for all physiologic measures and BMI. Figure 2 shows the adjusted differences in relative Heart Age between the alternative and full algorithms per gender and then per 10-year age categories. For all alternative algorithms, the overestimation of the relative Heart Age increased with age. For women, alternative algorithms overestimated relative Heart Age significantly more than for men, except for alternative algorithm #4, for which no gender effect was seen (*P*=.84). As shown in Figure 3, for all alternative algorithms, the overestimation of the relative Heart Age lessened with increasing CVD risk.

### Association Between Subject Characteristics and Awareness of Physiological Risk Factor Values

Adjusted odds ratios for the relations between users’ characteristics and their awareness of TC, HDL-C, and SBP are presented in Table 2. Relatively, age, total number of risk factors, and awareness of other physiological risk factors were the strongest predictors. Among men, awareness of HDL-C values was 26% (95% CI 25-27) higher, while awareness of SBP values was 20% (95% CI 79-80) lower, compared to women. For all three physiological risk factors, an increase in awareness was associated with increasing age (ie, 5-7% per additional year). Diabetes and family history of CVD were consistently associated with higher awareness of physiological risk factor values. Diabetics were 1.47-1.74 times more likely to know their values than non-diabetics, and awareness among users with a family history of CVD was 22-43% higher compared to users without a family history of CVD. Users with overweight or obesity were significantly more aware of their SBP values (OR 1.13, 95% CI 1.12-1.14 and OR 1.20, 95% CI 1.19-1.21) compared to normal weight users, but significantly less aware of their HDL-C values (OR 0.89, 95% CI .89-.91 and OR 0.91, 95% CI .73-.75).

Awareness of the three physiological risk factors decreased with increasing number of unhealthy CVD risk factors (by 41-47% per additional risk factor) and with increasing Heart Age (by 3-8% per additional Heart Age year). Among current smokers, awareness of TC, HDL-C, and SBP was 29-48% lower compared to non-smokers. Users with high SBP values were significantly less likely to be aware of their TC and HDL-C values compared to those with normal SBP, with OR 0.94 (95% CI 0.93-0.95) and OR 0.46 (95% CI 0.34-0.63) respectively. Using the exact value of a physiological measure as predicting variable, instead of classification into unhealthy or normal values, generally did not lead to a better fit of the logistic regression models. Awareness of the three physiological risk factor values was strongly interrelated (ie, users who knew one value were very likely to also know the other values). This was particularly the case for awareness of TC and HDL-C values; users who were aware of their TC were over 4000-fold more likely to also know their HDL-C and vice versa.

**Table 2:** Adjusted logistic regression examining the relations between users’ characteristics and their awareness of TC, HDL-C, and SBP values (N=2,744,091)^a^.

Characteristics		Awareness TC^b^		Awareness HDL-C^b^		Awareness SBP^b^	
		OR (95% CI)	R^2^	OR (95% CI)	R^2^	OR (95% CI)	R^2^
**Demographics**							
	Age, yrs	1.07 (1.07-1.07)	.164	1.06 (1.06-1.06)	.085	1.05 (1.05-1.05)	.123
	Gender, male	1.08 (1.08-1.09)	.000	1.26 (1.25-1.27)	.002	0.80 (0.79-0.80)	.003
**CVD risk estimate**							
	Heart Age, yrs^c^	0.97 (0.96-0.97)	.011	0.92 (0.92-0.92)	.042	0.97 (0.97-0.97)	.009
	10-yr CVD risk, %	0.96 (0.96-0.97)	.011	0.94 (0.94-0.94)	.020	1.02 (1.02-1.02)	.002
	Total unhealthy CVD risk factors, n	0.54 (0.54-0.54)	.110	0.59 (0.59-0.60)	.065	0.53 (0.53-0.53)	.141
**Risk factor prevalence**							
	Current smoker	0.67 (0.66-0.68)	.004	0.52 (0.52-0.53)	.007	0.71 (0.71-0.72)	.005
	Overweight, BMI≥25^d^	1.05 (1.04-1.06)	.000	0.90(0.89-0.91)	.001	1.13 (1.12-1.14)	.002
	Obese, BMI≥30^d^	1.03 (1.02-1.04)	.000	0.74 (0.73-0.75)	.001	1.20 (1.19-1.21)	.002
	Diabetic	1.74 (1.71-1.77)	.003	1.48 (1.45-1.51)	.001	1.47 (1.46-1.50)	.001
	High TC (≥240 mg/dl)^e^	—	—	1.07 (1.06-1.09)	.000	0.67 (0.66-0.68)	.004
	Low HDL-C (≤40 mg/dl)^f^	0.46 (0.34-0.63)	.007	—	—	1.05 (1.01-1.10)	.000
	High SBP (≥140 mmHg)^g^	0.94 (0.93-0.95)	.000	0.79 (0.77-0.80)	.001	—	—
	Family history of CVD	1.43 (1.42-1.44)	.006	1.26 (1.24-1.27)	.001	1.22 (1.22-1.23)	.002
**Awareness of physiological measures**							
	Awareness TC values	—	—	4364.73 (3,797.62-5,016.52)	.379	4.68 (4.65-4.72)	.069
	Awareness HDL-C values	4,125.41 (3,589.46-4,741.37)	.208	—	—	6.24 (6.13-3.34)	.027
	Awareness SBP values	4.85 (4.81-4.89)	.082	6.18 (6.08-6.29)	.056	—	—

^a^All logistic regressions were adjusted for age, country, and gender where appropriate. All ORs were significant (*P*<.001) with the exception of low HDL-C and awareness of SBP (*P*=.024).

^b^R^2^ shows the Nagelkerke R^2^ unique variance attributable to this variable after controlling for age, gender, and country where appropriate.

^c^Heart Age was calculated from different algorithms depending on available valid values of TC, HDL-C, and SBP.

^d^Users with valid BMI values (n=2,742,818), reference category is normal weight (BMI<25).

^e^Users with valid TC values (n=618,210).

^f^Users with valid HDL-C values (n=191,219).

^g^Users with valid SBP values (n=1,466,836).

##  Discussion

### Principal Results and Implications

The potential positive impact of Web-based health assessment tools on public health is highly dependent upon whether the tools reach the right audience and the quality of health assessment that is provided. This study evaluated whether a health assessment tool communicating CVD risk was reaching an audience with elevated risk, evaluated the impact of having correct input information (eg, awareness of key physiological risk factors), and examined whether certain subgroups were less likely to provide complete input information. Overall, the Heart Age tool was shown to reach the intended audience of people who are at risk of developing a CVD event, and it was demonstrated that missing information on physiological risk factors resulted in overestimation of (relative) Heart Age. Specific subgroups of people were indeed at higher risk for not providing complete risk factor information. The Heart Age tool reached on average users who were at moderate risk (men) and low risk (women); few users had a high risk (>20% CVD risk). Of note, the average Heart Age user had multiple CVD risk factors. Prevalence of smoking, overweight, and obesity among Heart Age users was within the usual ranges commonly observed in the studied countries [25-27]. However, rates of high TC, high SBP, and diabetes were lower [15,26,28,29]. This may be explained by the underrepresentation of people aged 60 years and older and to high rates of undiagnosed diabetes commonly seen in these populations [30-32]. Information bias may also play a role in the relatively low prevalence of high TC and SBP, as TC and SBP levels of those who were aware of their values may systematically differ from those who were not aware [33]. Others previously reported that specific population groups that are more likely to have high CVD risk, like older people, and those with preventable health problems or chronic conditions, and people of lower socioeconomic status are generally less likely to search for health information, possibly due to limited Internet access and usage [3,10,34,35] and relatively low eHealth literacy (ie, ability to seek, find, understand, and apply health information from electronic sources) [36]. The accessibility of Web-based health applications to these population groups at particularly high (absolute) CVD risk should therefore still be improved (eg, through increasing Internet access in public places, developing health apps for mobile use, using user friendly language, and targeted promotion).

The Heart Age tool was designed to reach people at risk of developing CVD, where interventions are still viable. The absolute risk of the current users was lower than expected, suggesting that the tool may be reaching a large proportion of “healthy” users. However, on average, these people had multiple unhealthy CVD risk factors, and their heart age was on average 4 years older than their real age. This suggests that their risk factors are elevated for their age, and they have high lifetime risk of developing CVD if nothing is done to improve their lifestyle. Therefore, the audience reached by Heart Age *was* the intended audience—a group that is a good target for disease prevention. This is of particular interest for younger people where a low absolute 10-year CVD risk can be falsely reassuring [37-40], especially in the presence of multiple raised risk factors. Heart Age was shown to be more likely perceived as a wake-up call among younger (30-45 year old) smokers and/or obese users [41]. Previous studies also showed that Heart Age motivated people to make healthy behavior changes or had an emotional impact [7,42,43], and led to greater reductions in cholesterol, blood pressure, and CVD risk than when a percentage for 10-year CVD risk was provided [42]. This highlights that health self-assessment tools, and particularly Heart Age, can be useful for health promotion at the population level.

A large proportion of Heart Age users did not know their TC, HDL-C, or SBP values and therefore received an overestimation of (relative) Heart Age that may cause inappropriate distress among users of the tool. This highlights the importance of complete risk factor information for reliable risk communication of Web-based health self-assessment tools. Therefore, Web-based health assessment tools should mention the potential impact of not knowing your risk factors and encourage users who have not recently been tested to get tested. As expected, the more physiological risk factors that were known, the more accurately (relative) Heart Age was predicted. Because awareness of TC alone did not add much to the accuracy of predicted Heart Age, cholesterol tests should generally measure both TC and HDL-C concentrations. Furthermore, the use of proxy-measures to estimate lacking physiological risk factor values in Web-based health applications should be explored and validated. These could include questions on whether people are diagnosed with hypertension or hypercholesterolemia or have been prescribed cholesterol-lowering medication. The Heart Age tool is continuously improved based on current learnings, and its algorithms are fine-tuned to increase its accuracy; this may include space for new measurements that improve the performance of the tool but also important variations that may be necessary for different countries or ethnic groups. The finding that overestimation of Heart Age diminished with increasing CVD risk is presumably attributable to the capping of Heart Age. Precautionary measures to restrict extreme outcomes of Web-based health assessment tools may generally be useful to limit large inaccuracies in predicted disease risk. Withholding people who have incomplete risk factor information from accessing Web-based self-assessment tools may be a missed opportunity as the Heart Age tool has been shown to motivate women to get their cholesterol tested [41].

Smokers and people with several unhealthy CVD risk factors were particularly at risk of not being aware of their (other) physiological CVD risk factors. Therefore, if health self-assessment tools are targeted at these groups, special attention should be paid to a thorough inventory of all relevant risk factors, a careful communication of the estimated disease risk within some confidence limits, and compelling encouragement to get to know one’s risk factor values. As awareness of physiological CVD risk factors by itself has been shown to motivate people to take preventive actions [44,45], awareness of risk factor values should generally be promoted among smokers and those with other known CVD risk factors. This also aligns with the general policy in CVD risk prevention, which encourages looking at the combination of all CVD risk factors [22,46]. The finding that awareness of physiological risk factors decreased with increasing Heart Age needs to be considered with care as this may be partly explained by Heart Age being overestimated when risk factor information is missing. The number of total CVD risk factors explained 6.5-14.1% of the variation in awareness of risk factors, indicating that other characteristics beyond risk factors may be more important predictors of awareness of TC, HDL-C, and SBP.

### Strengths and Limitations

The current study was based on a large sample size of almost three million Heart Age tool users, providing confidence in findings. Moreover, the sample was drawn from 13 countries, enhancing the generalizability of results. However, there are some limitations that should be taken into account when interpreting the results. First, the data are self-reported and not verified for accuracy through objective measures; this is particularly an issue for physiological data, which are not always accurately recalled or may not be from a recent measurement. Second, the impact of awareness of physiological risk factors on Heart Age was estimated based on a relatively small and healthy subsample of Heart Age users; therefore, this finding may not be generalizable. Thus, while from this study the impact of missing risk factor information is shown to be a concern, future research is required to investigate the effect of awareness of physiological CVD risk factors on Heart Age in a sample where self-reported information on awareness of physiological risk factor values is combined with objective measurements of all Heart Age tool parameters.

### Conclusions

Heart Age is a Web-based CVD risk communication tool, which was found on average to reach people with multiple elevated CVD risk factors and a Heart Age higher than their real age. This highlights that Web-based self -assessment health tools can be a useful means to interact with people who are at risk of developing disease, and where interventions are still viable. Missing information in the self-assessment health tools was shown to result in inaccurate self-health assessments. This demonstrates the importance of complete input information into health self-assessment tools and the need to encourage people who do not know their values to get tested. Specific subgroups of people were found to be particularly at risk for not having complete input information required for health self-assessment tools, and as such, more care needs to be taken if health self-assessment tools are being deployed to these groups. Extra efforts need to be made to raise awareness in these subgroups.

## Multimedia Appendix 1

Screenshots of the Heart Age tool.

## Multimedia Appendix 2

Full and alternative algorithms used to calculate 10-year CVD risk and Heart Age.

## Abbreviations

ANCOVA: analysis of covariance
BMI: body mass index
CVD: cardiovascular disease
HDL-C: high density lipoprotein cholesterol
TC: total cholesterol
SBP: systolic blood pressure
