# Supplementary figures and images for: Web-Based Self-Assessment Health Tools: Who Are the Users and What Is the Impact of Missing Input Information?
Source: J Med Internet Res. 2014 Sep 26;16(9):e215. doi: 10.2196/jmir.3146 (PMC4211033; doi:10.2196/jmir.3146)

## Slide 1
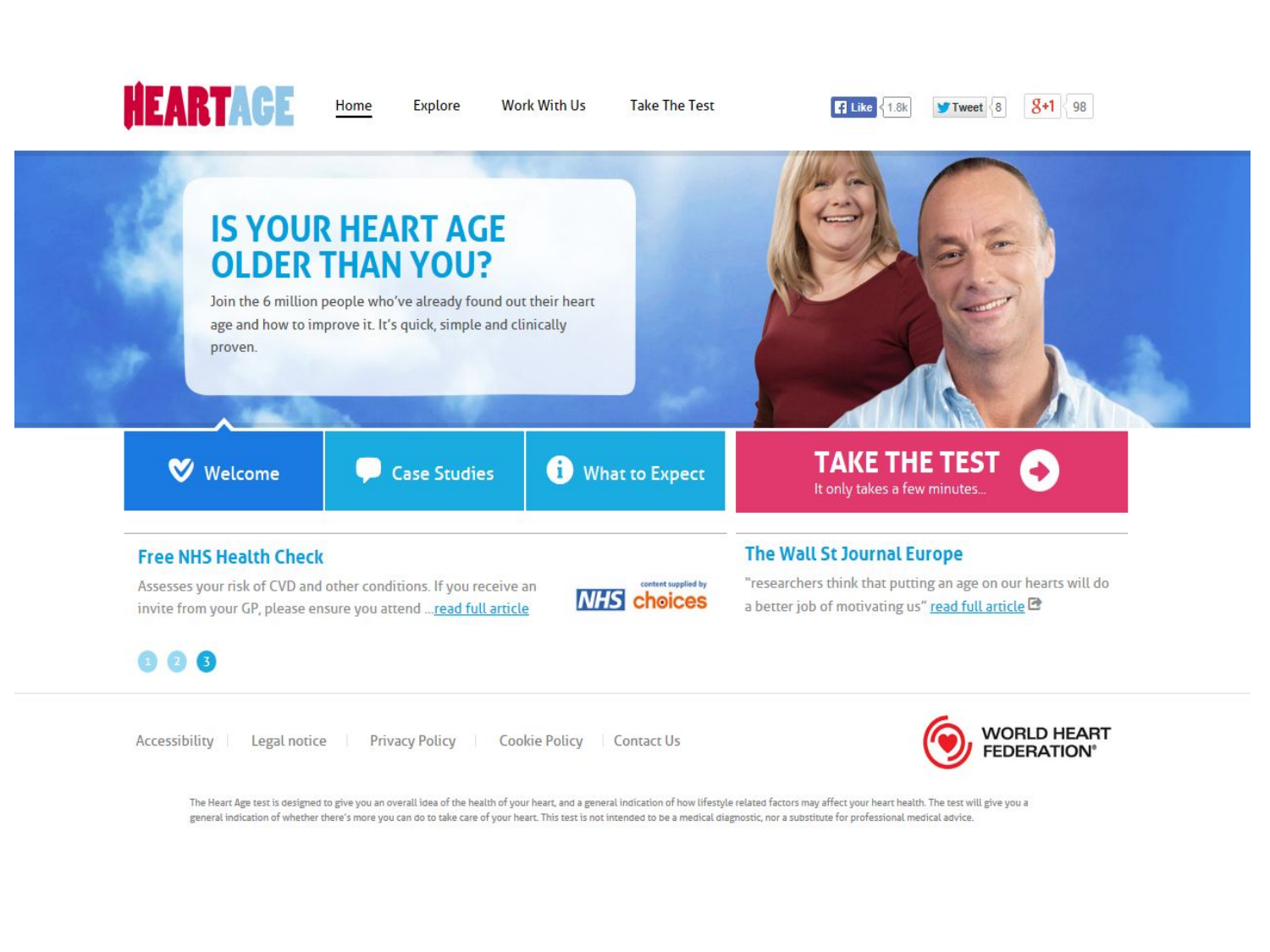

## Slide 2
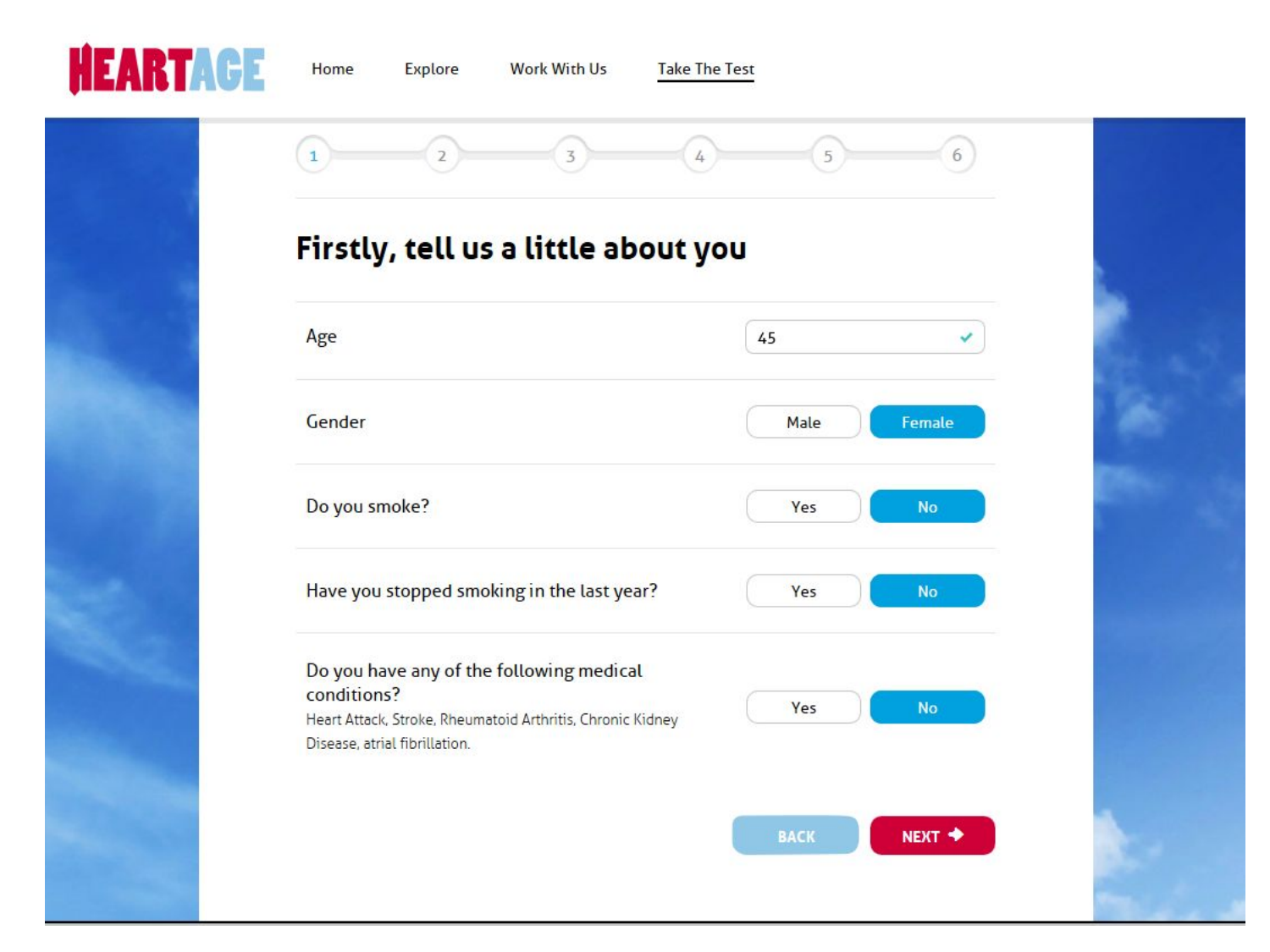

## Slide 3
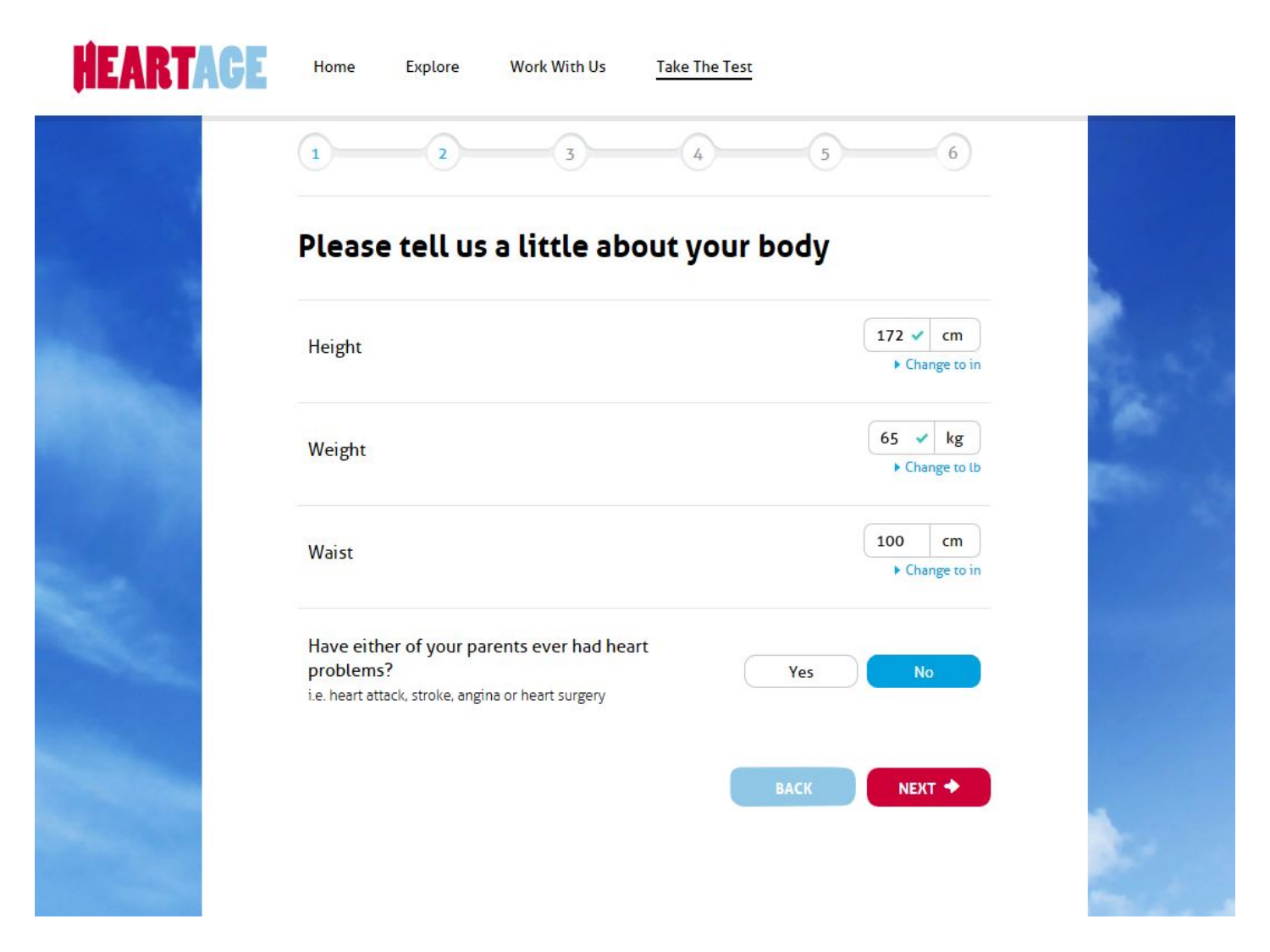

## Slide 4
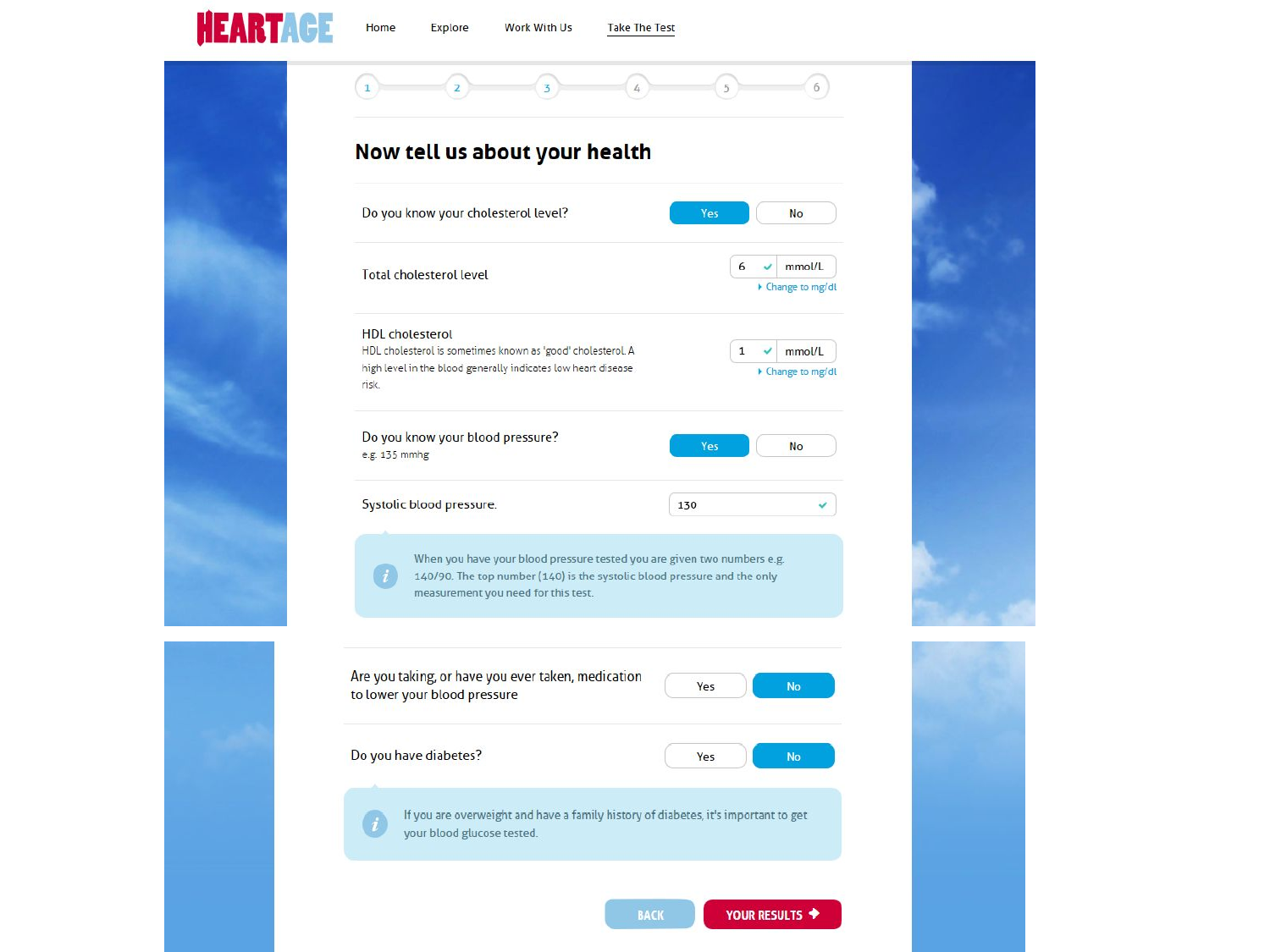

## Slide 5
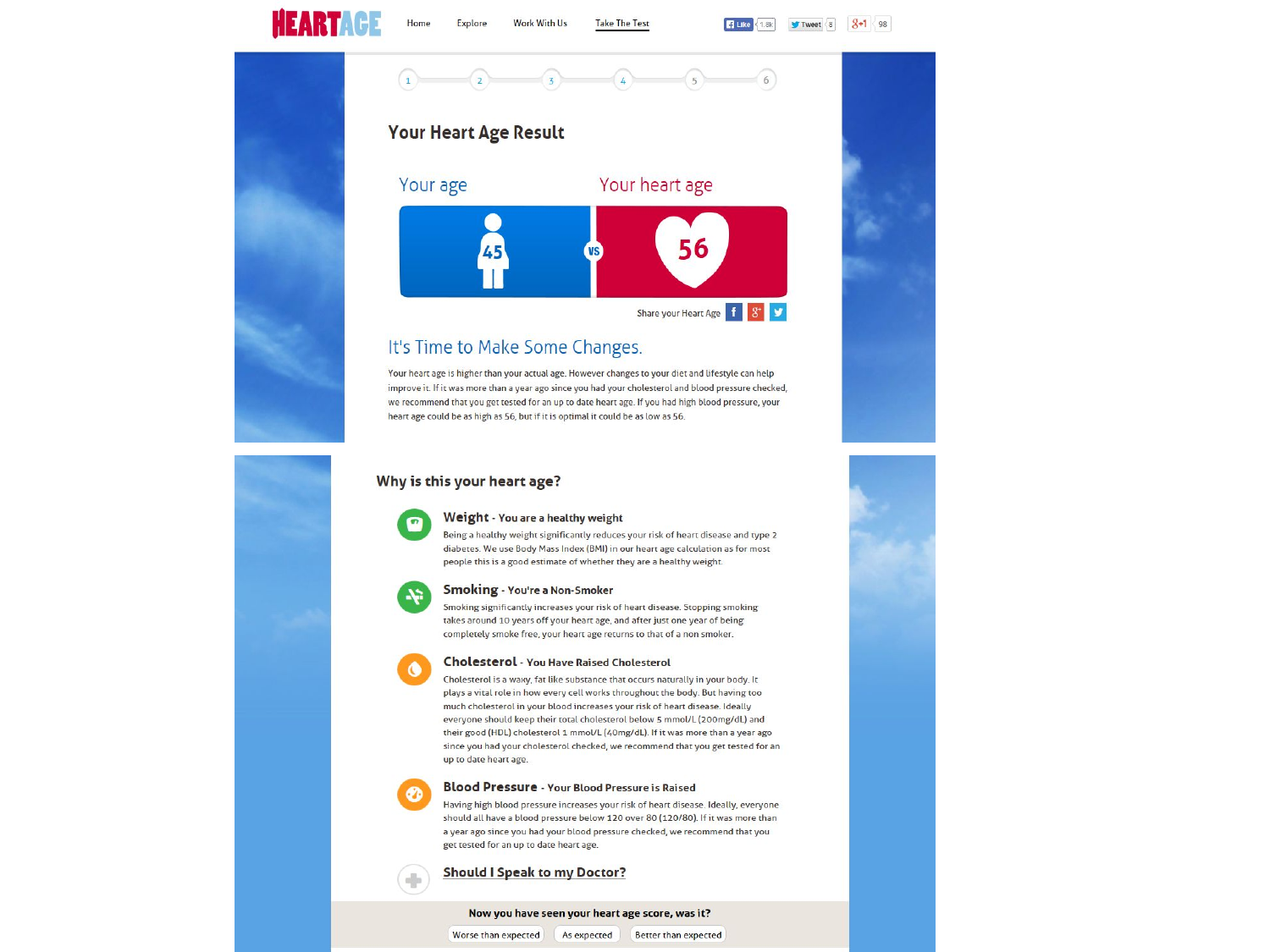

Supplement: Supplementary file 1 [file jmir_v16i9e215_app1.pptx]
