# Supplementary material for: Web-Based Self-Assessment Health Tools: Who Are the Users and What Is the Impact of Missing Input Information?
Source: J Med Internet Res. 2014 Sep 26;16(9):e215. doi: 10.2196/jmir.3146 (PMC4211033; doi:10.2196/jmir.3146)
Supplement: Supplementary file 2 [file jmir_v16i9e215_app2.pdf]

Heart Age Algorithms

Where

TOT = Total Cholesterol (mg/dl)
HDL = HDL Cholesterol (mg/dl?)
DIAB = 1 if prevalent diabetes, 0 otherwise
SMK = 1 if smoking in previous year, 0 otherwise
SBP = Systolic Blood Pressure (mmHg)
“Ln” = natural logarithm

FULL ALGORITHM *(TC, HDL-C & SBP known)*

→ ***Calculate 10-year CVD Risk :***

Men on Antihypertensive Rx:

[illegible]

Men not on Antihypertensive Rx:

[illegible]

Women on Antihypertensive Rx:

[illegible]

Women not on Antihypertensive Rx:

[illegible]

→ ***Calculate Heart Age :***

[illegible]

Men < 60 years:

[illegible]

Men >= 60 years:

Women < 60 years:

[illegible]

Women >= 60 years:

[illegible]

ALTERNATIVE ALGORITHM #1 *(TC & HDL-C known, SBP unknown)*

→ ***Calculate 10-year CVD Risk:***

Men:

[illegible]

Women:

[illegible]

→ ***Calculate Heart Age:***

[illegible]

Men:

Women:

[illegible]

ALTERNATIVE ALGORITHM #2 *(TC, SBP, BMI known, HDL unknown)*

→ ***Calculate 10-year CVD Risk:***

Men on Antihypertensive Rx:

[illegible]

Men not on Antihypertensive Rx:

[illegible]

Women on Antihypertensive Rx:

[illegible]

Women not on Antihypertensive Rx:

[illegible]

→ ***Calculate Heart Age:***

Men <60 years:

[illegible]

Men >= 60 years:

[illegible]

Women < 60 years:

[illegible]

Women >= 60 years:

[illegible]

note. In 2013 the reference value for TC was increased to 200 to increase the sensitivity

ALTERNATIVE ALGORITHM #3 *(TC, BMI known, HDL & SBP unknown)*

→ *Calculate 10-year CVD Risk:*

[illegible]

Men:

Women:

[illegible]

→ *Calculate Heart Age:*

[illegible]

Men:

[illegible]

Women:

ALTERNATIVE ALGORITHM #4 (*SBP & BMI known, TC & HDL cholesterol unknown)*

→ ***Calculate 10-year CVD Risk:***

Men on Antihypertensive Rx:

[illegible]

Men not on Antihypertensive Rx:

[illegible]

Women on Antihypertensive Rx:

[illegible]

Women not on Antihypertensive Rx:

[illegible]

→ ***Calculate Heart Age:***

Men < 60 years of age:

[illegible]

Men >= 60 years of age:

[illegible]

Women < 60 years of age:

[illegible]

Women >= 60 years of age:

[illegible]

ALTERNATIVE ALGORITHM #5 (*BMI known, SBP, TC & HDL cholesterol unknown)*

→ ***Calculate 10-year CVD Risk:***

[illegible]

Men:

[illegible]

Women:

→ ***Calculate Heart Age:***

Men:

[illegible]

Women:

[illegible]
